# P-2207. HCV Reinfection is Associated with Cocaine Use and Ongoing Injection Drug Use in People with OUD: Outcomes from the ANCHOR and LOOP Studies

**DOI:** 10.1093/ofid/ofae631.2361

**Published:** 2025-01-29

**Authors:** Tina Liu, Habib Omari, Emade Ebah, Rahwa Eyasu, Onyinyechi Ogbumbadiugha-Weekes, Amelia Cover, Ashley Davis, Meredith Zoltick, Rachel Silk, Meghan Derenoncourt, Dorcas Salifu, Sabina Ghale, Phyllis Bijole, Miriam Jones, Randy Kier, David Sternberg, Sarah Kattakuzhy, Elana S Rosenthal

**Affiliations:** National Institutes of Health - Critical Care Medicine Department, Durham, North Carolina; University of Maryland Baltimore, Baltimore, Maryland; Institute for Human Virology (IHV), University of Maryland School of Medicine, Washington, District of Columbia; Institute for Human Virology (IHV), University of Maryland School of Medicine, Washington, District of Columbia; Institute of Human Virology, University of Maryland School of Medicine, Baltimore, Maryland; Institute of Human Virology/University of Maryland School of Medicine, New York, New York; Institute for Human Virology (IHV), University of Maryland School of Medicine, Washington, District of Columbia; University of Maryland Baltimore - Institute of Human Virology, Baltimore, Maryland; University of Maryland, Washington, DC; University of Maryland, Baltimore, Baltimore, Maryland; University of Maryland Baltimore - Institute of Human Virology, Baltimore, Maryland; Institute of Human Virology/University of Maryland School of Medicine, New York, New York; HIPS.org, Washington, District of Columbia; HIPS.org, Washington, District of Columbia; HIPS, Washington, DC; University of Maryland Baltimore - Institute of Human Virology, Baltimore, Maryland; Institute for Human Virology (IHV), University of Maryland School of Medicine, Washington, District of Columbia; Institute for Human Virology (IHV), University of Maryland School of Medicine, Washington, District of Columbia

## Abstract

**Background:**

Despite achieving sustained virologic response (SVR), people with HCV, opioid use disorder (OUD) and ongoing injection drug use (IDU) remain at risk of reinfection. Identification and retreatment of reinfected individuals is critical to HCV elimination. We sought to evaluate the rate of HCV reinfection, factors associated with reinfection, and retreatment in a cohort of people who inject drugs (PWID).

**Table 1.**
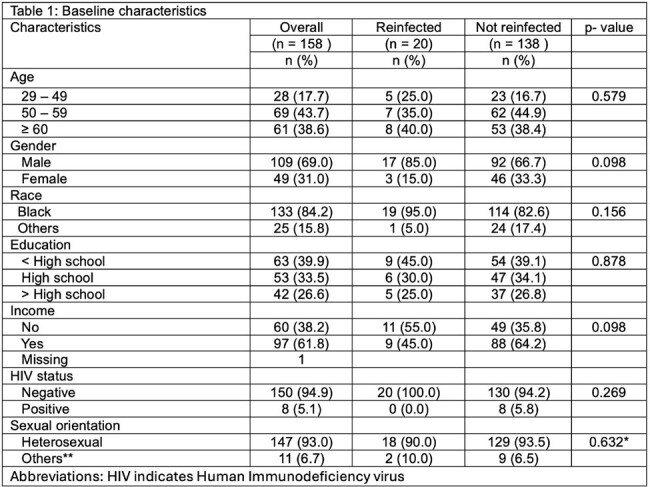
Bivariate analysis of baseline sociodemographic characteristics.

**Methods:**

The ANCHOR study provided HCV treatment for 198 people with chronic HCV, OUD, and recent opioid use. Those who achieved SVR underwent screening for HCV reinfection, with retreatment initiated per standard of care. At each visit, surveys were administered to evaluate ongoing risk behaviors and medication for OUD (MOUD) status. Patients were followed for 96 weeks with optional enrollment in a 2-year extension study (LOOP).

**Table 2.**
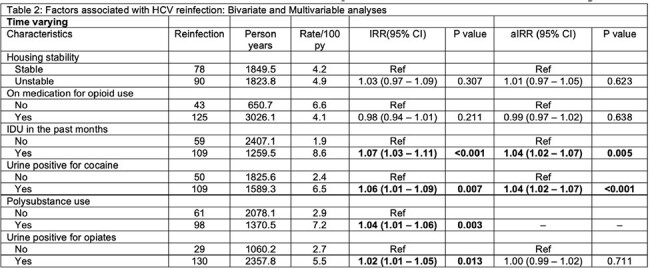
Bivariate (IRR) and multivariable (aIRR) analyses of factors associated with HCV reinfection.

**Results:**

158 individuals achieved SVR and were followed after treatment completion for 353 person-years, median 90 weeks (IQR 84-160 weeks). Subjects were predominantly male (69%), Black (84.2%), middle-aged (58 years), and HIV-negative (94.9%). Twenty individuals (12.7%) were reinfected a median of 77 weeks (IQR 60-105 weeks) after HCV treatment, at a rate of 5.7/100 person-years. Reinfection was associated with cocaine use (p< 0.001) and IDU in the past month (p=0.005).

Of the 20 reinfected individuals, 16 (80%) initiated HCV re-treatment a median of 26 weeks after detection (IQR 4-64 weeks), with 12 (75%) achieving SVR, 1 currently on treatment, and 5 deaths.

**Figure 1.**
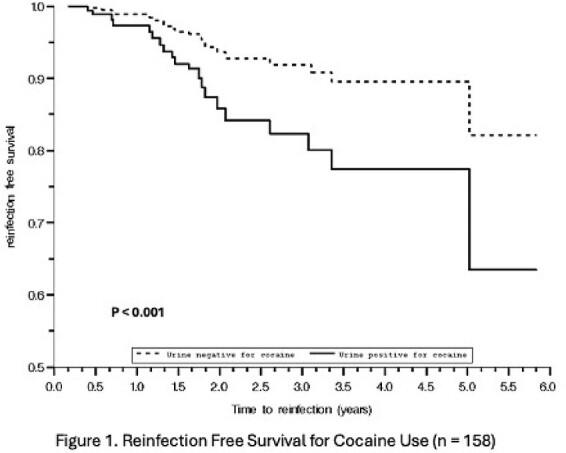
Kaplan-Meier reinfection free survival curve assessing associations of cocaine use with HCV reinfection.

**Conclusion:**

In this cohort of people with OUD recently cured of HCV, we observed moderate rates of HCV reinfection associated with cocaine use and past month IDU, with high rates of retreatment uptake and SVR. These data highlight that in people with active drug use, longitudinal follow-up for retesting and retreatment is critical for HCV elimination. Enhanced accessibility to and engagement with harm-reduction services – such as syringe service programs - and interventions specifically addressing polysubstance use are crucial to reducing ongoing HCV transmission and reinfection.

**Figure:**
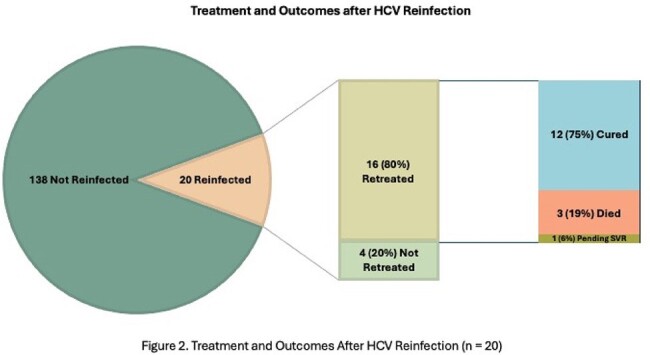

**Disclosures:**

Elana S. Rosenthal, MD, Gilead Sciences: Grant/Research Support|Merck: Grant/Research Support

